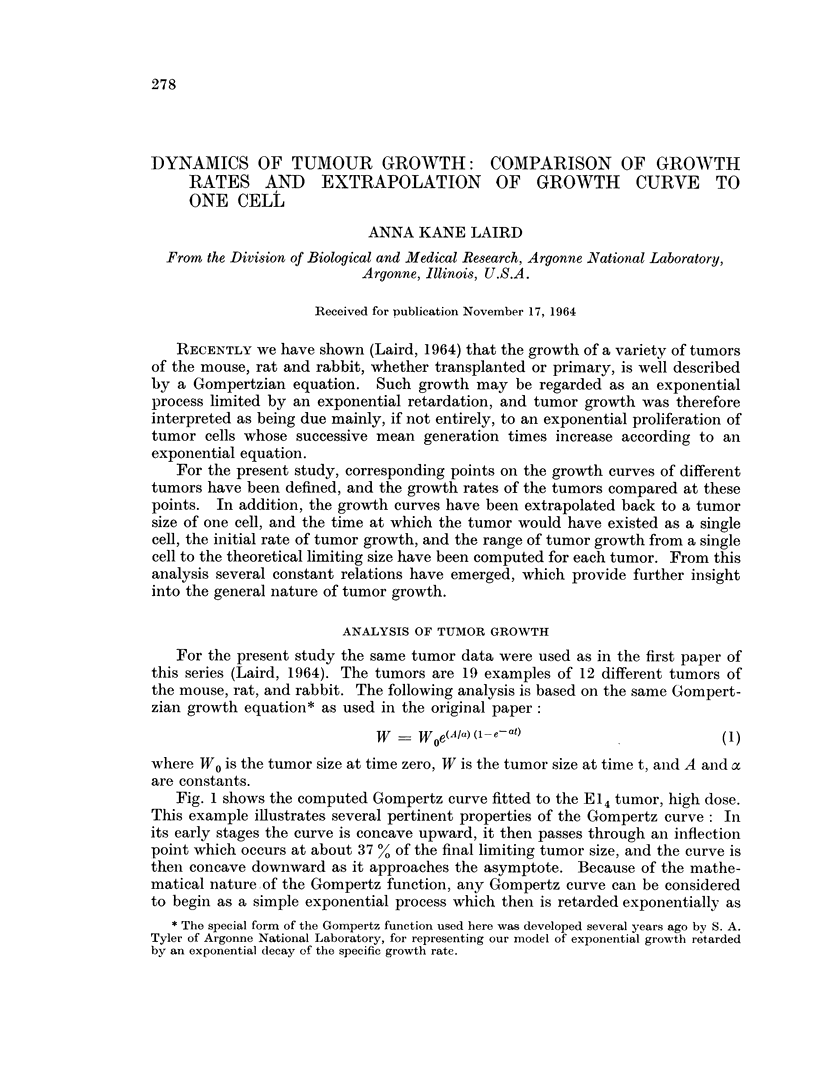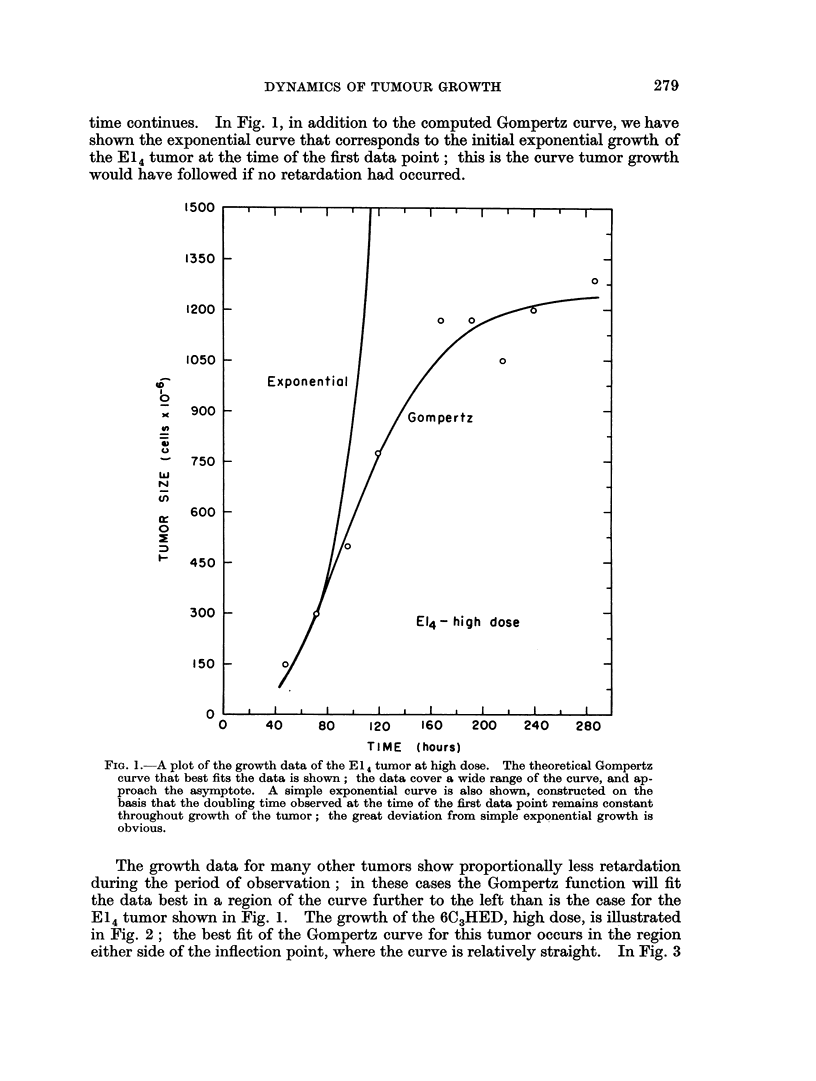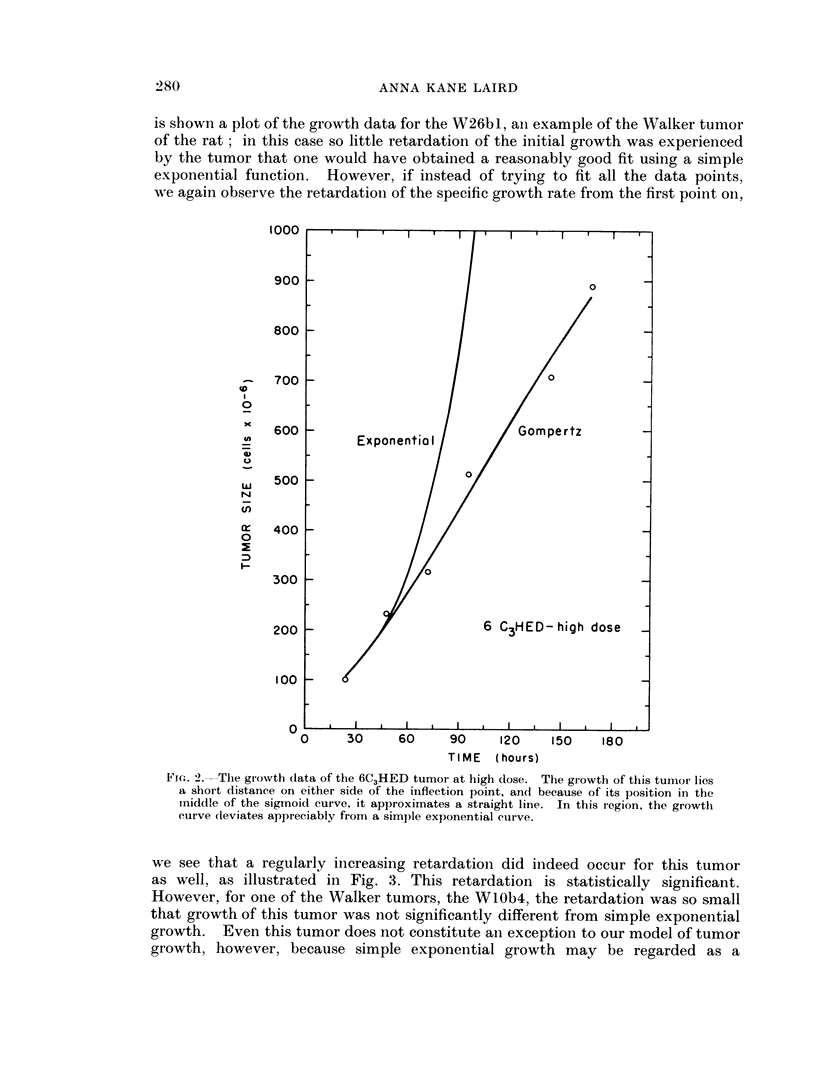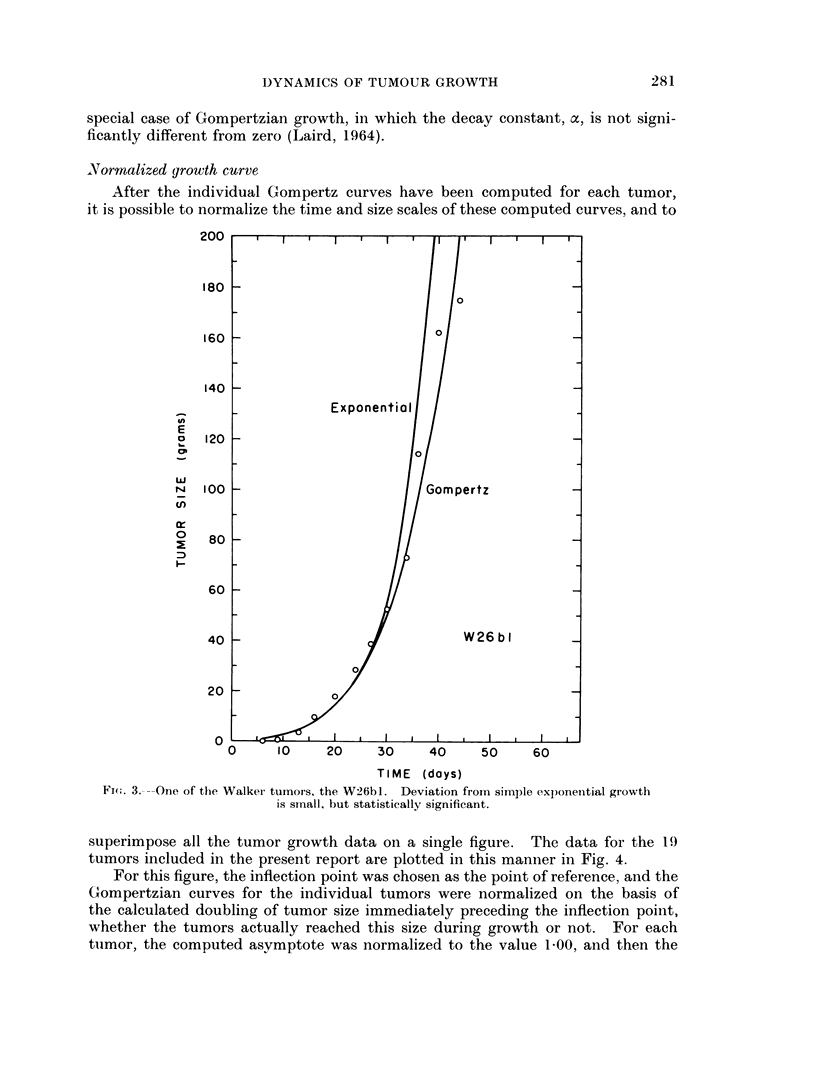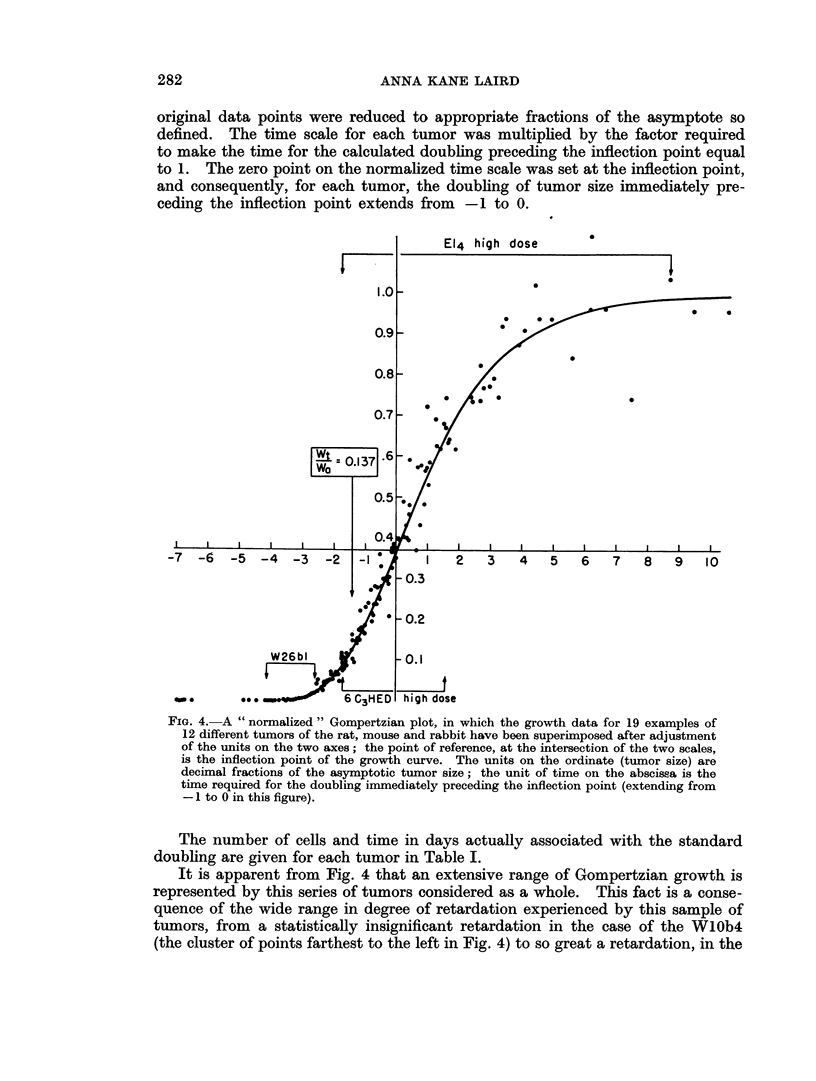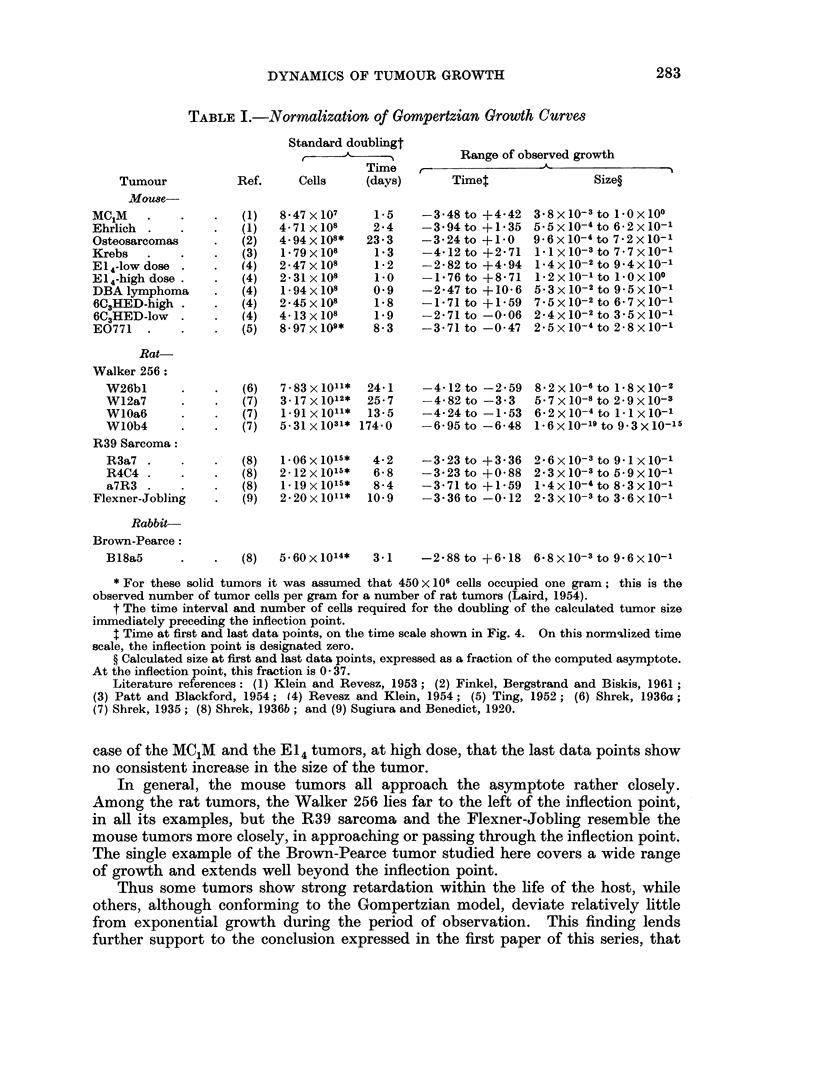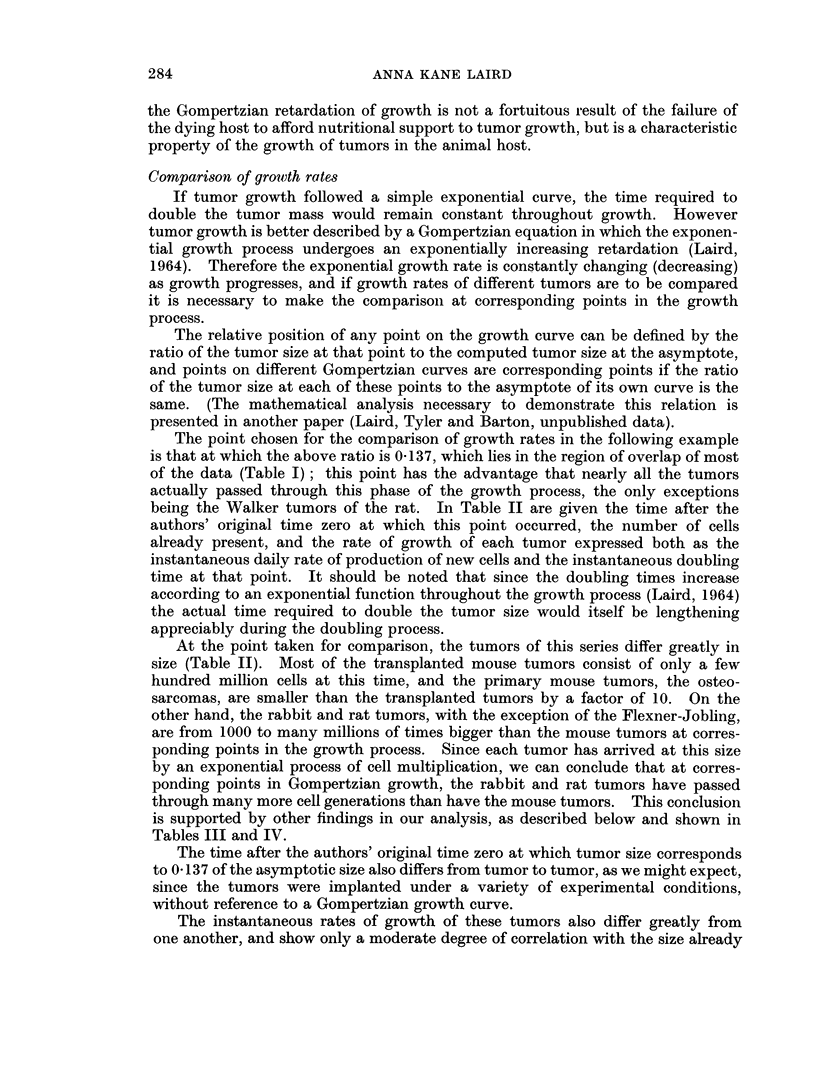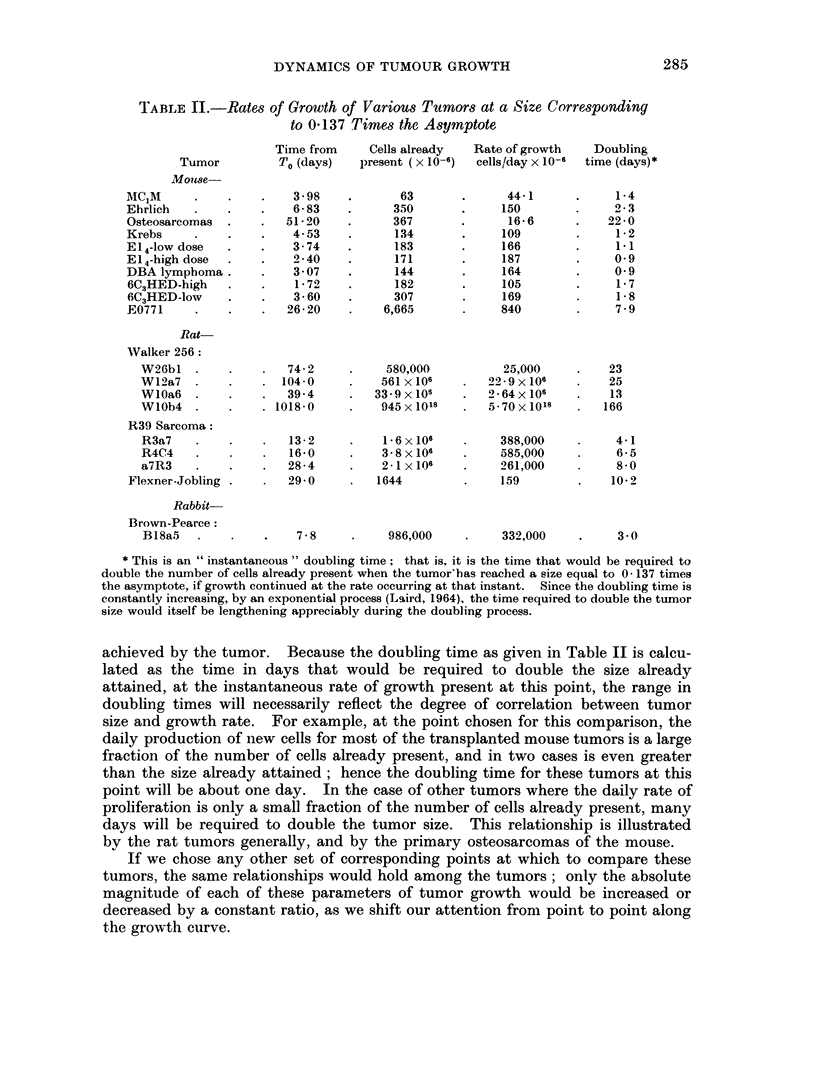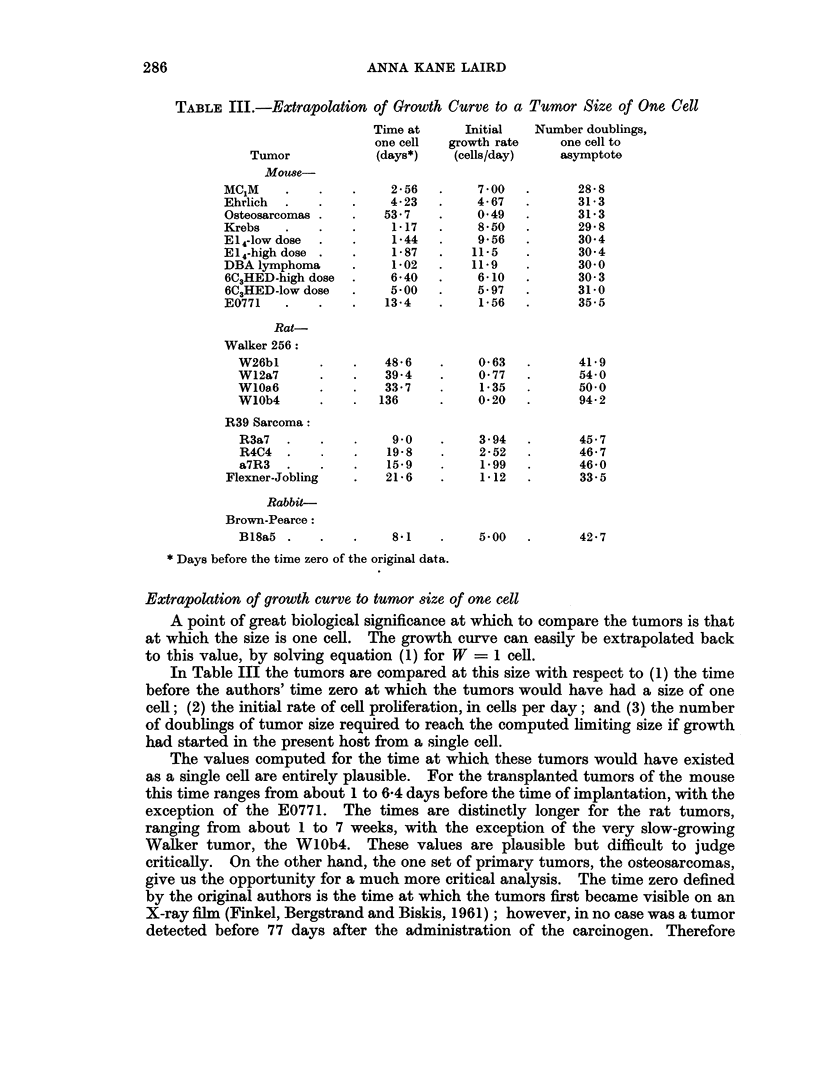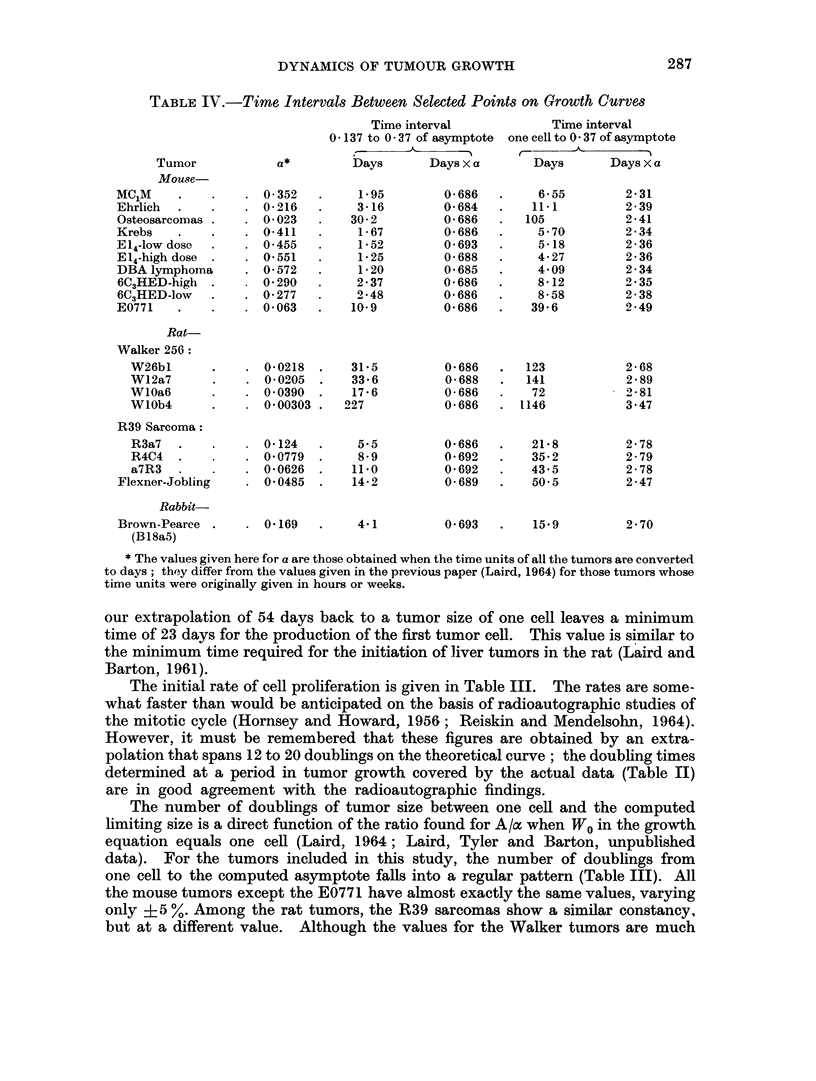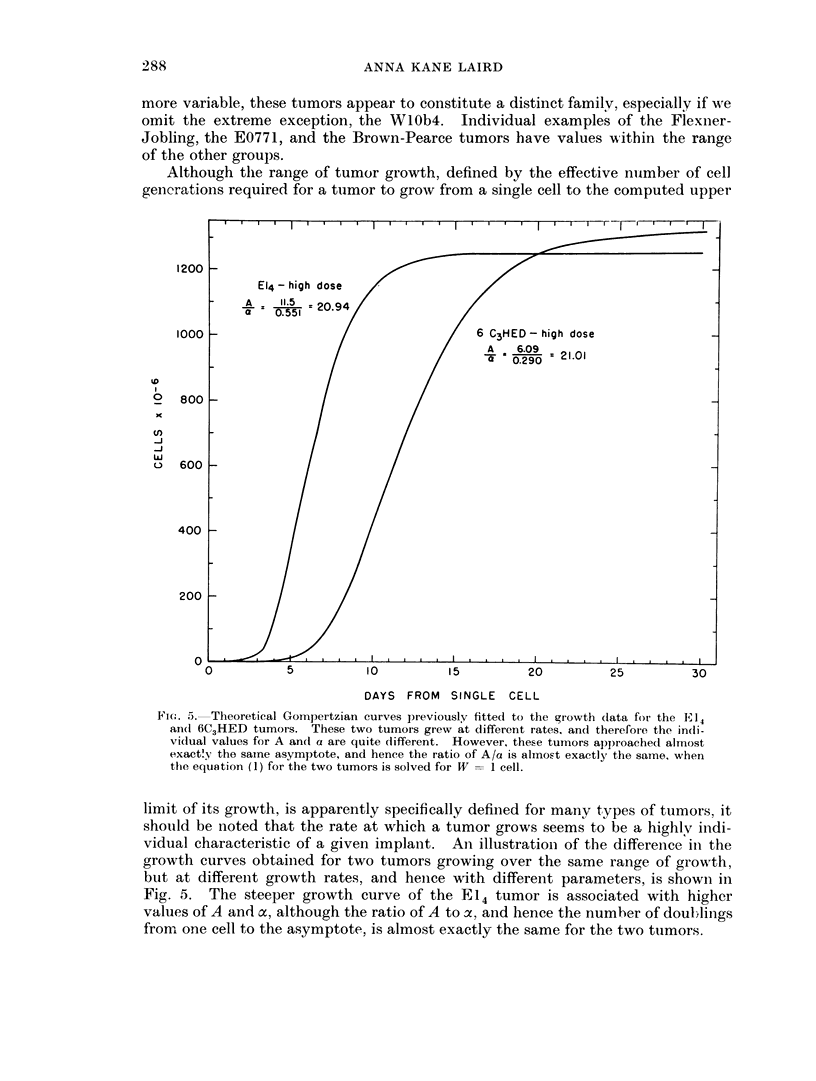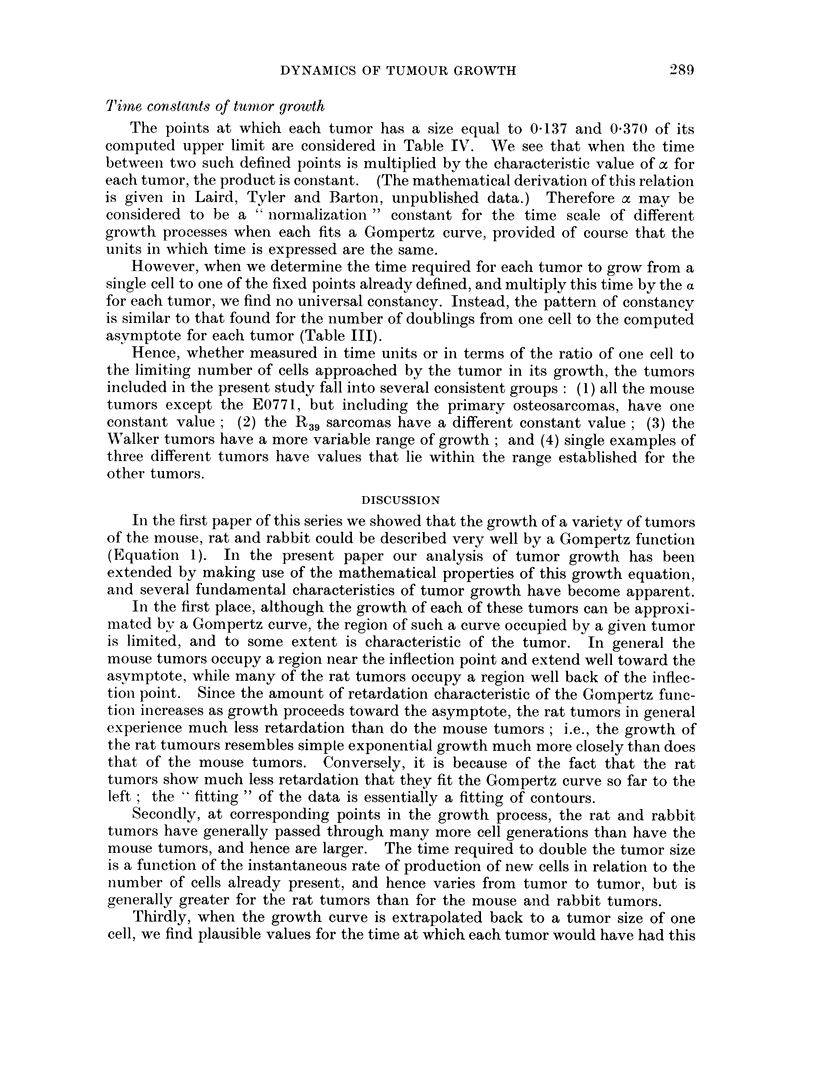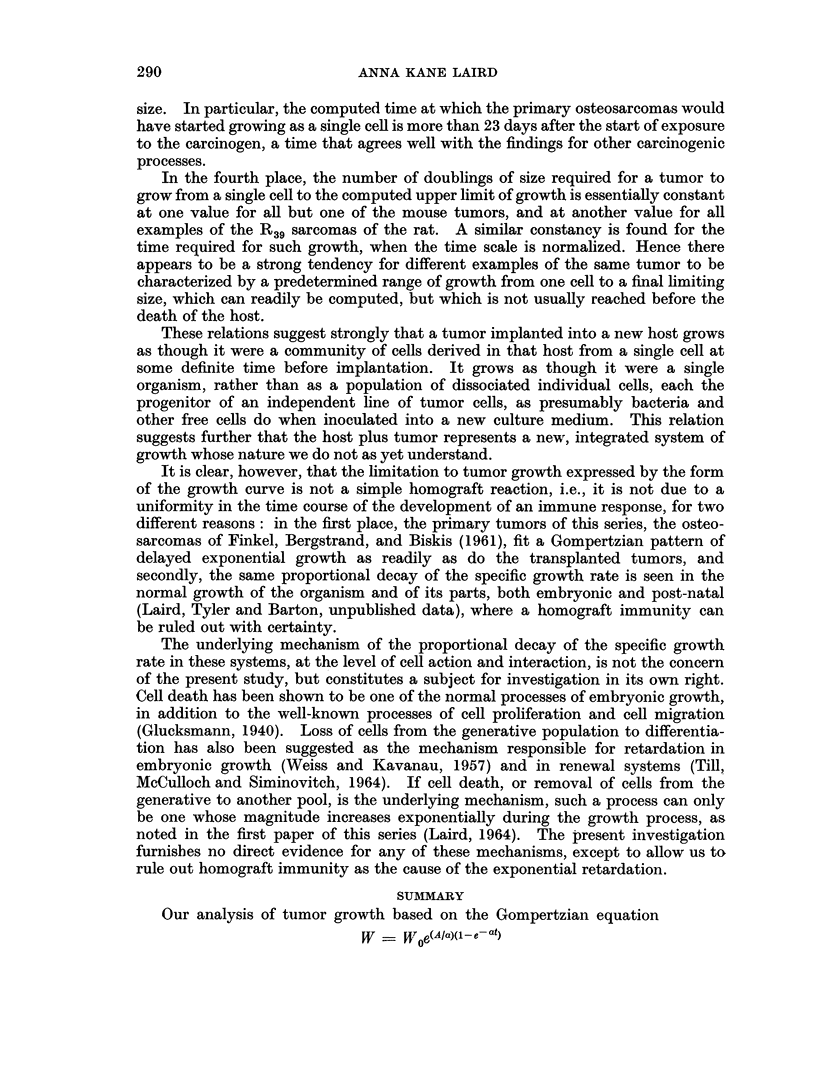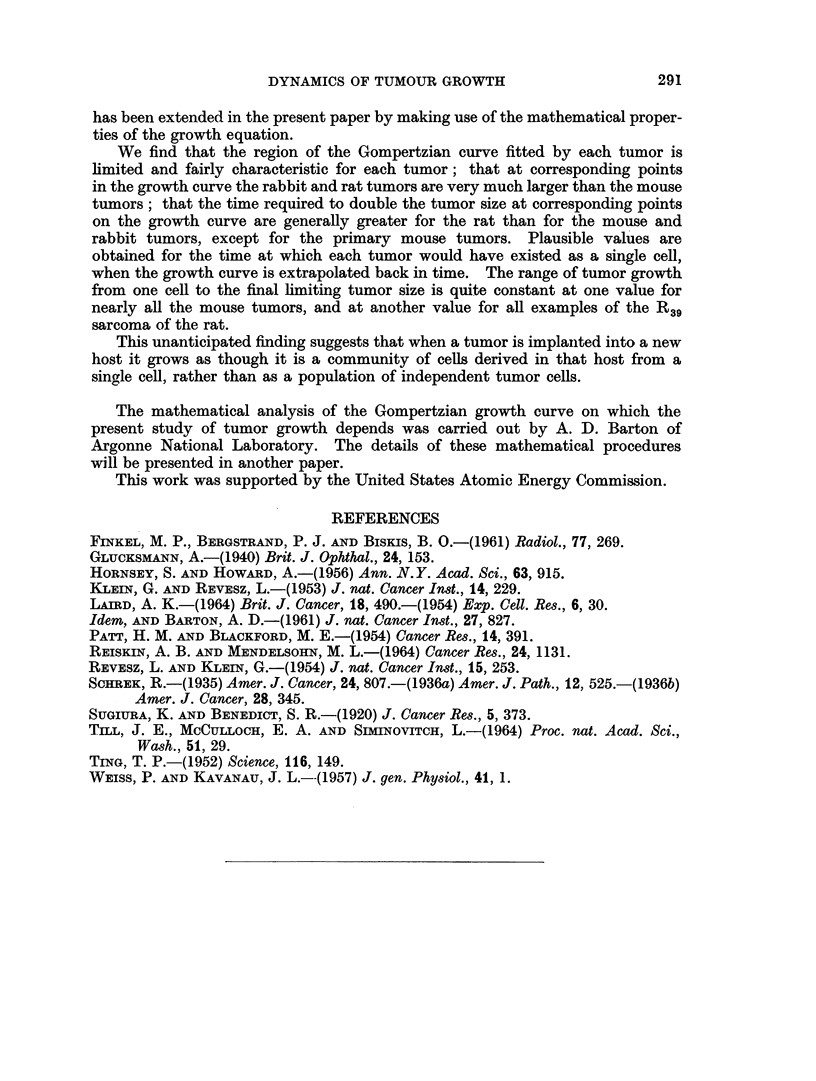# Dynamics of Tumour Growth: Comparison of Growth Rates and Extrapolation of Growth Curve to One Cell

**DOI:** 10.1038/bjc.1965.32

**Published:** 1965-06

**Authors:** Anna Kane Laird


### DYNAMICS OF TUMOUR GROWTH: COMPARISON OF GROWTH RATES AND EXTRAPOLATION OF GROWTH CURVE TO ONE CELL

ANNA KANE LAIRD

*From the Division of Biological and Medical Research, Argonne National Laboratory, Argonne, Illinois, U.S.A.*

Received for publication November 17, 1964

RECENTLY we have shown (Laird, 1964) that the growth of a variety of tumors of the mouse, rat and rabbit, whether transplanted or primary, is well described by a Gompertzian equation. Such growth may be regarded as an exponential process limited by an exponential retardation, and tumor growth was therefore interpreted as being due mainly, if not entirely, to an exponential proliferation of tumor cells whose successive mean generation times increase according to an exponential equation.

For the present study, corresponding points on the growth curves of different tumors have been defined, and the growth rates of the tumors compared at these points. In addition, the growth curves have been extrapolated back to a tumor size of one cell, and the time at which the tumor would have existed as a single cell, the initial rate of tumor growth, and the range of tumor growth from a single cell to the theoretical limiting size have been computed for each tumor. From this analysis several constant relations have emerged, which provide further insight into the general nature of tumor growth.

### ANALYSIS OF TUMOR GROWTH

For the present study the same tumor data were used as in the first paper of this series (Laird, 1964). The tumors are 19 examples of 12 different tumors of the mouse, rat, and rabbit. The following analysis is based on the same Gompertzian growth equation* as used in the original paper :

$$W = W_0 e^{(A/a)(1-e^{-at})} \qquad (1)$$

where $W_0$ is the tumor size at time zero, $W$ is the tumor size at time t, and $A$ and $\alpha$ are constants.

Fig. 1 shows the computed Gompertz curve fitted to the $El_4$ tumor, high dose. This example illustrates several pertinent properties of the Gompertz curve : In its early stages the curve is concave upward, it then passes through an inflection point which occurs at about 37 % of the final limiting tumor size, and the curve is then concave downward as it approaches the asymptote. Because of the mathematical nature of the Gompertz function, any Gompertz curve can be considered to begin as a simple exponential process which then is retarded exponentially as

* The special form of the Gompertz function used here was developed several years ago by S. A. Tyler of Argonne National Laboratory, for representing our model of exponential growth retarded by an exponential decay of the specific growth rate.

time continues. In Fig. 1, in addition to the computed Gompertz curve, we have shown the exponential curve that corresponds to the initial exponential growth of the $El_4$ tumor at the time of the first data point; this is the curve tumor growth would have followed if no retardation had occurred.

FIG. 1.—A plot of the growth data of the $El_4$ tumor at high dose. The theoretical Gompertz curve that best fits the data is shown; the data cover a wide range of the curve, and approach the asymptote. A simple exponential curve is also shown, constructed on the basis that the doubling time observed at the time of the first data point remains constant throughout growth of the tumor; the great deviation from simple exponential growth is obvious.

The growth data for many other tumors show proportionally less retardation during the period of observation; in these cases the Gompertz function will fit the data best in a region of the curve further to the left than is the case for the $El_4$ tumor shown in Fig. 1. The growth of the $6C_3HED$, high dose, is illustrated in Fig. 2; the best fit of the Gompertz curve for this tumor occurs in the region either side of the inflection point, where the curve is relatively straight. In Fig. 3

is shown a plot of the growth data for the W26b1, an example of the Walker tumor of the rat ; in this case so little retardation of the initial growth was experienced by the tumor that one would have obtained a reasonably good fit using a simple exponential function. However, if instead of trying to fit all the data points, we again observe the retardation of the specific growth rate from the first point on,

FIG. 2.—The growth data of the $6C_3HED$ tumor at high dose. The growth of this tumor lies a short distance on either side of the inflection point, and because of its position in the middle of the sigmoid curve, it approximates a straight line. In this region, the growth curve deviates appreciably from a simple exponential curve.

we see that a regularly increasing retardation did indeed occur for this tumor as well, as illustrated in Fig. 3. This retardation is statistically significant. However, for one of the Walker tumors, the W10b4, the retardation was so small that growth of this tumor was not significantly different from simple exponential growth. Even this tumor does not constitute an exception to our model of tumor growth, however, because simple exponential growth may be regarded as a special case of Gompertzian growth, in which the decay constant, $\alpha$, is not significantly different from zero (Laird, 1964).

*Normalized growth curve*

After the individual Gompertz curves have been computed for each tumor, it is possible to normalize the time and size scales of these computed curves, and to

FIG. 3.—One of the Walker tumors, the W26bl. Deviation from simple exponential growth is small, but statistically significant.

superimpose all the tumor growth data on a single figure. The data for the 19 tumors included in the present report are plotted in this manner in Fig. 4.

For this figure, the inflection point was chosen as the point of reference, and the Gompertzian curves for the individual tumors were normalized on the basis of the calculated doubling of tumor size immediately preceding the inflection point, whether the tumors actually reached this size during growth or not. For each tumor, the computed asymptote was normalized to the value 1·00, and then the original data points were reduced to appropriate fractions of the asymptote so defined. The time scale for each tumor was multiplied by the factor required to make the time for the calculated doubling preceding the inflection point equal to 1. The zero point on the normalized time scale was set at the inflection point, and consequently, for each tumor, the doubling of tumor size immediately preceding the inflection point extends from —1 to 0.

FIG. 4.—A " normalized " Gompertzian plot, in which the growth data for 19 examples of 12 different tumors of the rat, mouse and rabbit have been superimposed after adjustment of the units on the two axes; the point of reference, at the intersection of the two scales, is the inflection point of the growth curve. The units on the ordinate (tumor size) are decimal fractions of the asymptotic tumor size; the unit of time on the abscissa is the time required for the doubling immediately preceding the inflection point (extending from —1 to 0 in this figure).

The number of cells and time in days actually associated with the standard doubling are given for each tumor in Table I.

It is apparent from Fig. 4 that an extensive range of Gompertzian growth is represented by this series of tumors considered as a whole. This fact is a consequence of the wide range in degree of retardation experienced by this sample of tumors, from a statistically insignificant retardation in the case of the W10b4 (the cluster of points farthest to the left in Fig. 4) to so great a retardation, in the

TABLE I.—*Normalization of Gompertzian Growth Curves*

| Tumour | Ref. | Standard doubling† Cells | Standard doubling† Time (days) | Range of observed growth Time‡ | Range of observed growth Size§ |
|---|---|---|---|---|---|
| *Mouse*— | | | | | |
| $MC_1M$ | (1) | $8 \cdot 47 \times 10^7$ | 1·5 | −3·48 to +4·42 | $3 \cdot 8 \times 10^{-3}$ to $1 \cdot 0 \times 10^0$ |
| Ehrlich | (1) | $4 \cdot 71 \times 10^8$ | 2·4 | −3·94 to +1·35 | $5 \cdot 5 \times 10^{-4}$ to $6 \cdot 2 \times 10^{-1}$ |
| Osteosarcomas | (2) | $4 \cdot 94 \times 10^8$* | 23·3 | −3·24 to +1·0 | $9 \cdot 6 \times 10^{-4}$ to $7 \cdot 2 \times 10^{-1}$ |
| Krebs | (3) | $1 \cdot 79 \times 10^8$ | 1·3 | −4·12 to +2·71 | $1 \cdot 1 \times 10^{-3}$ to $7 \cdot 7 \times 10^{-1}$ |
| $El_4$-low dose | (4) | $2 \cdot 47 \times 10^8$ | 1·2 | −2·82 to +4·94 | $1 \cdot 4 \times 10^{-2}$ to $9 \cdot 4 \times 10^{-1}$ |
| $El_4$-high dose | (4) | $2 \cdot 31 \times 10^8$ | 1·0 | −1·76 to +8·71 | $1 \cdot 2 \times 10^{-1}$ to $1 \cdot 0 \times 10^0$ |
| DBA lymphoma | (4) | $1 \cdot 94 \times 10^8$ | 0·9 | −2·47 to +10·6 | $5 \cdot 3 \times 10^{-2}$ to $9 \cdot 5 \times 10^{-1}$ |
| $6C_3HED$-high | (4) | $2 \cdot 45 \times 10^8$ | 1·8 | −1·71 to +1·59 | $7 \cdot 5 \times 10^{-2}$ to $6 \cdot 7 \times 10^{-1}$ |
| $6C_3HED$-low | (4) | $4 \cdot 13 \times 10^8$ | 1·9 | −2·71 to −0·06 | $2 \cdot 4 \times 10^{-2}$ to $3 \cdot 5 \times 10^{-1}$ |
| EO771 | (5) | $8 \cdot 97 \times 10^9$* | 8·3 | −3·71 to −0·47 | $2 \cdot 5 \times 10^{-4}$ to $2 \cdot 8 \times 10^{-1}$ |
| *Rat*— | | | | | |
| Walker 256 : | | | | | |
| W26b1 | (6) | $7 \cdot 83 \times 10^{11}$* | 24·1 | −4·12 to −2·59 | $8 \cdot 2 \times 10^{-6}$ to $1 \cdot 8 \times 10^{-2}$ |
| W12a7 | (7) | $3 \cdot 17 \times 10^{12}$* | 25·7 | −4·82 to −3·3 | $5 \cdot 7 \times 10^{-8}$ to $2 \cdot 9 \times 10^{-3}$ |
| W10a6 | (7) | $1 \cdot 91 \times 10^{11}$* | 13·5 | −4·24 to −1·53 | $6 \cdot 2 \times 10^{-4}$ to $1 \cdot 1 \times 10^{-1}$ |
| W10b4 | (7) | $5 \cdot 31 \times 10^{31}$* | 174·0 | −6·95 to −6·48 | $1 \cdot 6 \times 10^{-19}$ to $9 \cdot 3 \times 10^{-15}$ |
| R39 Sarcoma : | | | | | |
| R3a7 | (8) | $1 \cdot 06 \times 10^{15}$* | 4·2 | −3·23 to +3·36 | $2 \cdot 6 \times 10^{-3}$ to $9 \cdot 1 \times 10^{-1}$ |
| R4C4 | (8) | $2 \cdot 12 \times 10^{15}$* | 6·8 | −3·23 to +0·88 | $2 \cdot 3 \times 10^{-3}$ to $5 \cdot 9 \times 10^{-1}$ |
| a7R3 | (8) | $1 \cdot 19 \times 10^{15}$* | 8·4 | −3·71 to +1·59 | $1 \cdot 4 \times 10^{-4}$ to $8 \cdot 3 \times 10^{-1}$ |
| Flexner-Jobling | (9) | $2 \cdot 20 \times 10^{11}$* | 10·9 | −3·36 to −0·12 | $2 \cdot 3 \times 10^{-3}$ to $3 \cdot 6 \times 10^{-1}$ |
| *Rabbit*— | | | | | |
| Brown-Pearce : | | | | | |
| B18a5 | (8) | $5 \cdot 60 \times 10^{14}$* | 3·1 | −2·88 to +6·18 | $6 \cdot 8 \times 10^{-3}$ to $9 \cdot 6 \times 10^{-1}$ |

* For these solid tumors it was assumed that $450 \times 10^6$ cells occupied one gram ; this is the observed number of tumor cells per gram for a number of rat tumors (Laird, 1954).

† The time interval and number of cells required for the doubling of the calculated tumor size immediately preceding the inflection point.

‡ Time at first and last data points, on the time scale shown in Fig. 4. On this normalized time scale, the inflection point is designated zero.

§ Calculated size at first and last data points, expressed as a fraction of the computed asymptote. At the inflection point, this fraction is 0·37.

Literature references : (1) Klein and Revesz, 1953 ; (2) Finkel, Bergstrand and Biskis, 1961 ; (3) Patt and Blackford, 1954 ; (4) Revesz and Klein, 1954 ; (5) Ting, 1952 ; (6) Shrek, 1936*a* ; (7) Shrek, 1935 ; (8) Shrek, 1936*b* ; and (9) Sugiura and Benedict, 1920.

case of the $MC_1M$ and the $El_4$ tumors, at high dose, that the last data points show no consistent increase in the size of the tumor.

In general, the mouse tumors all approach the asymptote rather closely. Among the rat tumors, the Walker 256 lies far to the left of the inflection point, in all its examples, but the R39 sarcoma and the Flexner-Jobling resemble the mouse tumors more closely, in approaching or passing through the inflection point. The single example of the Brown-Pearce tumor studied here covers a wide range of growth and extends well beyond the inflection point.

Thus some tumors show strong retardation within the life of the host, while others, although conforming to the Gompertzian model, deviate relatively little from exponential growth during the period of observation. This finding lends further support to the conclusion expressed in the first paper of this series, that the Gompertzian retardation of growth is not a fortuitous result of the failure of the dying host to afford nutritional support to tumor growth, but is a characteristic property of the growth of tumors in the animal host.

*Comparison of growth rates*

If tumor growth followed a simple exponential curve, the time required to double the tumor mass would remain constant throughout growth. However tumor growth is better described by a Gompertzian equation in which the exponential growth process undergoes an exponentially increasing retardation (Laird, 1964). Therefore the exponential growth rate is constantly changing (decreasing) as growth progresses, and if growth rates of different tumors are to be compared it is necessary to make the comparison at corresponding points in the growth process.

The relative position of any point on the growth curve can be defined by the ratio of the tumor size at that point to the computed tumor size at the asymptote, and points on different Gompertzian curves are corresponding points if the ratio of the tumor size at each of these points to the asymptote of its own curve is the same. (The mathematical analysis necessary to demonstrate this relation is presented in another paper (Laird, Tyler and Barton, unpublished data).

The point chosen for the comparison of growth rates in the following example is that at which the above ratio is 0·137, which lies in the region of overlap of most of the data (Table I); this point has the advantage that nearly all the tumors actually passed through this phase of the growth process, the only exceptions being the Walker tumors of the rat. In Table II are given the time after the authors' original time zero at which this point occurred, the number of cells already present, and the rate of growth of each tumor expressed both as the instantaneous daily rate of production of new cells and the instantaneous doubling time at that point. It should be noted that since the doubling times increase according to an exponential function throughout the growth process (Laird, 1964) the actual time required to double the tumor size would itself be lengthening appreciably during the doubling process.

At the point taken for comparison, the tumors of this series differ greatly in size (Table II). Most of the transplanted mouse tumors consist of only a few hundred million cells at this time, and the primary mouse tumors, the osteosarcomas, are smaller than the transplanted tumors by a factor of 10. On the other hand, the rabbit and rat tumors, with the exception of the Flexner-Jobling, are from 1000 to many millions of times bigger than the mouse tumors at corresponding points in the growth process. Since each tumor has arrived at this size by an exponential process of cell multiplication, we can conclude that at corresponding points in Gompertzian growth, the rabbit and rat tumors have passed through many more cell generations than have the mouse tumors. This conclusion is supported by other findings in our analysis, as described below and shown in Tables III and IV.

The time after the authors' original time zero at which tumor size corresponds to 0·137 of the asymptotic size also differs from tumor to tumor, as we might expect, since the tumors were implanted under a variety of experimental conditions, without reference to a Gompertzian growth curve.

The instantaneous rates of growth of these tumors also differ greatly from one another, and show only a moderate degree of correlation with the size already

TABLE II.—*Rates of Growth of Various Tumors at a Size Corresponding to 0·137 Times the Asymptote*

| Tumor | Time from $T_0$ (days) | Cells already present ($\times 10^{-6}$) | Rate of growth cells/day $\times 10^{-6}$ | Doubling time (days)* |
|---|---|---|---|---|
| *Mouse*— | | | | |
| $MC_1M$ | 3·98 | 63 | 44·1 | 1·4 |
| Ehrlich | 6·83 | 350 | 150 | 2·3 |
| Osteosarcomas | 51·20 | 367 | 16·6 | 22·0 |
| Krebs | 4·53 | 134 | 109 | 1·2 |
| $El_4$-low dose | 3·74 | 183 | 166 | 1·1 |
| $El_4$-high dose | 2·40 | 171 | 187 | 0·9 |
| DBA lymphoma | 3·07 | 144 | 164 | 0·9 |
| $6C_3HED$-high | 1·72 | 182 | 105 | 1·7 |
| $6C_3HED$-low | 3·60 | 307 | 169 | 1·8 |
| E0771 | 26·20 | 6,665 | 840 | 7·9 |
| *Rat*— | | | | |
| Walker 256 : | | | | |
| W26b1 | 74·2 | 580,000 | 25,000 | 23 |
| W12a7 | 104·0 | $561 \times 10^6$ | $22·9 \times 10^6$ | 25 |
| W10a6 | 39·4 | $33·9 \times 10^6$ | $2·64 \times 10^6$ | 13 |
| W10b4 | 1018·0 | $945 \times 10^{18}$ | $5·70 \times 10^{18}$ | 166 |
| R39 Sarcoma : | | | | |
| R3a7 | 13·2 | $1·6 \times 10^6$ | 388,000 | 4·1 |
| R4C4 | 16·0 | $3·8 \times 10^6$ | 585,000 | 6·5 |
| a7R3 | 28·4 | $2·1 \times 10^6$ | 261,000 | 8·0 |
| Flexner-Jobling | 29·0 | 1644 | 159 | 10·2 |
| *Rabbit*— | | | | |
| Brown-Pearce : | | | | |
| B18a5 | 7·8 | 986,000 | 332,000 | 3·0 |

* This is an " instantaneous " doubling time ; that is, it is the time that would be required to double the number of cells already present when the tumor has reached a size equal to 0·137 times the asymptote, if growth continued at the rate occurring at that instant. Since the doubling time is constantly increasing, by an exponential process (Laird, 1964), the time required to double the tumor size would itself be lengthening appreciably during the doubling process.

achieved by the tumor. Because the doubling time as given in Table II is calculated as the time in days that would be required to double the size already attained, at the instantaneous rate of growth present at this point, the range in doubling times will necessarily reflect the degree of correlation between tumor size and growth rate. For example, at the point chosen for this comparison, the daily production of new cells for most of the transplanted mouse tumors is a large fraction of the number of cells already present, and in two cases is even greater than the size already attained ; hence the doubling time for these tumors at this point will be about one day. In the case of other tumors where the daily rate of proliferation is only a small fraction of the number of cells already present, many days will be required to double the tumor size. This relationship is illustrated by the rat tumors generally, and by the primary osteosarcomas of the mouse.

If we chose any other set of corresponding points at which to compare these tumors, the same relationships would hold among the tumors ; only the absolute magnitude of each of these parameters of tumor growth would be increased or decreased by a constant ratio, as we shift our attention from point to point along the growth curve.

TABLE III.—*Extrapolation of Growth Curve to a Tumor Size of One Cell*

| Tumor | Time at one cell (days*) | Initial growth rate (cells/day) | Number doublings, one cell to asymptote |
|---|---|---|---|
| *Mouse—* | | | |
| $MC_1M$ | 2·56 | 7·00 | 28·8 |
| Ehrlich | 4·23 | 4·67 | 31·3 |
| Osteosarcomas | 53·7 | 0·49 | 31·3 |
| Krebs | 1·17 | 8·50 | 29·8 |
| $El_4$-low dose | 1·44 | 9·56 | 30·4 |
| $El_4$-high dose | 1·87 | 11·5 | 30·4 |
| DBA lymphoma | 1·02 | 11·9 | 30·0 |
| $6C_3HED$-high dose | 6·40 | 6·10 | 30·3 |
| $6C_3HED$-low dose | 5·00 | 5·97 | 31·0 |
| E0771 | 13·4 | 1·56 | 35·5 |
| *Rat—* | | | |
| Walker 256 : | | | |
| W26b1 | 48·6 | 0·63 | 41·9 |
| W12a7 | 39·4 | 0·77 | 54·0 |
| W10a6 | 33·7 | 1·35 | 50·0 |
| W10b4 | 136 | 0·20 | 94·2 |
| R39 Sarcoma : | | | |
| R3a7 | 9·0 | 3·94 | 45·7 |
| R4C4 | 19·8 | 2·52 | 46·7 |
| a7R3 | 15·9 | 1·99 | 46·0 |
| Flexner-Jobling | 21·6 | 1·12 | 33·5 |
| *Rabbit—* | | | |
| Brown-Pearce : | | | |
| B18a5 | 8·1 | 5·00 | 42·7 |

* Days before the time zero of the original data.

*Extrapolation of growth curve to tumor size of one cell*

A point of great biological significance at which to compare the tumors is that at which the size is one cell. The growth curve can easily be extrapolated back to this value, by solving equation (1) for $W = 1$ cell.

In Table III the tumors are compared at this size with respect to (1) the time before the authors' time zero at which the tumors would have had a size of one cell; (2) the initial rate of cell proliferation, in cells per day; and (3) the number of doublings of tumor size required to reach the computed limiting size if growth had started in the present host from a single cell.

The values computed for the time at which these tumors would have existed as a single cell are entirely plausible. For the transplanted tumors of the mouse this time ranges from about 1 to 6·4 days before the time of implantation, with the exception of the E0771. The times are distinctly longer for the rat tumors, ranging from about 1 to 7 weeks, with the exception of the very slow-growing Walker tumor, the W10b4. These values are plausible but difficult to judge critically. On the other hand, the one set of primary tumors, the osteosarcomas, give us the opportunity for a much more critical analysis. The time zero defined by the original authors is the time at which the tumors first became visible on an X-ray film (Finkel, Bergstrand and Biskis, 1961); however, in no case was a tumor detected before 77 days after the administration of the carcinogen. Therefore

TABLE IV.—*Time Intervals Between Selected Points on Growth Curves*

| Tumor | $a$* | Time interval 0·137 to 0·37 of asymptote | | Time interval one cell to 0·37 of asymptote | |
|---|---|---|---|---|---|
| | | Days | Days × $a$ | Days | Days × $a$ |
| *Mouse—* | | | | | |
| $MC_1M$ | 0·352 | 1·95 | 0·686 | 6·55 | 2·31 |
| Ehrlich | 0·216 | 3·16 | 0·684 | 11·1 | 2·39 |
| Osteosarcomas | 0·023 | 30·2 | 0·686 | 105 | 2·41 |
| Krebs | 0·411 | 1·67 | 0·686 | 5·70 | 2·34 |
| $El_4$-low dose | 0·455 | 1·52 | 0·693 | 5·18 | 2·36 |
| $El_4$-high dose | 0·551 | 1·25 | 0·688 | 4·27 | 2·36 |
| DBA lymphoma | 0·572 | 1·20 | 0·685 | 4·09 | 2·34 |
| $6C_3HED$-high | 0·290 | 2·37 | 0·686 | 8·12 | 2·35 |
| $6C_3HED$-low | 0·277 | 2·48 | 0·686 | 8·58 | 2·38 |
| E0771 | 0·063 | 10·9 | 0·686 | 39·6 | 2·49 |
| *Rat—* | | | | | |
| Walker 256 : | | | | | |
| W26b1 | 0·0218 | 31·5 | 0·686 | 123 | 2·68 |
| W12a7 | 0·0205 | 33·6 | 0·688 | 141 | 2·89 |
| W10a6 | 0·0390 | 17·6 | 0·686 | 72 | 2·81 |
| W10b4 | 0·00303 | 227 | 0·686 | 1146 | 3·47 |
| R39 Sarcoma : | | | | | |
| R3a7 | 0·124 | 5·5 | 0·686 | 21·8 | 2·78 |
| R4C4 | 0·0779 | 8·9 | 0·692 | 35·2 | 2·79 |
| a7R3 | 0·0626 | 11·0 | 0·692 | 43·5 | 2·78 |
| Flexner-Jobling | 0·0485 | 14·2 | 0·689 | 50·5 | 2·47 |
| *Rabbit—* | | | | | |
| Brown-Pearce (B18a5) | 0·169 | 4·1 | 0·693 | 15·9 | 2·70 |

* The values given here for $a$ are those obtained when the time units of all the tumors are converted to days ; they differ from the values given in the previous paper (Laird, 1964) for those tumors whose time units were originally given in hours or weeks.

our extrapolation of 54 days back to a tumor size of one cell leaves a minimum time of 23 days for the production of the first tumor cell. This value is similar to the minimum time required for the initiation of liver tumors in the rat (Laird and Barton, 1961).

The initial rate of cell proliferation is given in Table III. The rates are somewhat faster than would be anticipated on the basis of radioautographic studies of the mitotic cycle (Hornsey and Howard, 1956 ; Reiskin and Mendelsohn, 1964). However, it must be remembered that these figures are obtained by an extrapolation that spans 12 to 20 doublings on the theoretical curve ; the doubling times determined at a period in tumor growth covered by the actual data (Table II) are in good agreement with the radioautographic findings.

The number of doublings of tumor size between one cell and the computed limiting size is a direct function of the ratio found for $A/\alpha$ when $W_0$ in the growth equation equals one cell (Laird, 1964 ; Laird, Tyler and Barton, unpublished data). For the tumors included in this study, the number of doublings from one cell to the computed asymptote falls into a regular pattern (Table III). All the mouse tumors except the E0771 have almost exactly the same values, varying only $\pm 5\%$. Among the rat tumors, the R39 sarcomas show a similar constancy, but at a different value. Although the values for the Walker tumors are much more variable, these tumors appear to constitute a distinct family, especially if we omit the extreme exception, the W10b4. Individual examples of the Flexner-Jobling, the E0771, and the Brown-Pearce tumors have values within the range of the other groups.

Although the range of tumor growth, defined by the effective number of cell generations required for a tumor to grow from a single cell to the computed upper

FIG. 5.—Theoretical Gompertzian curves previously fitted to the growth data for the $El_4$ and $6C_3HED$ tumors. These two tumors grew at different rates, and therefore the individual values for A and $a$ are quite different. However, these tumors approached almost exactly the same asymptote, and hence the ratio of $A/a$ is almost exactly the same, when the equation (1) for the two tumors is solved for $W = 1$ cell.

limit of its growth, is apparently specifically defined for many types of tumors, it should be noted that the rate at which a tumor grows seems to be a highly individual characteristic of a given implant. An illustration of the difference in the growth curves obtained for two tumors growing over the same range of growth, but at different growth rates, and hence with different parameters, is shown in Fig. 5. The steeper growth curve of the $El_4$ tumor is associated with higher values of $A$ and $\alpha$, although the ratio of $A$ to $\alpha$, and hence the number of doublings from one cell to the asymptote, is almost exactly the same for the two tumors.

*Time constants of tumor growth*

The points at which each tumor has a size equal to 0·137 and 0·370 of its computed upper limit are considered in Table IV. We see that when the time between two such defined points is multiplied by the characteristic value of $\alpha$ for each tumor, the product is constant. (The mathematical derivation of this relation is given in Laird, Tyler and Barton, unpublished data.) Therefore $\alpha$ may be considered to be a " normalization " constant for the time scale of different growth processes when each fits a Gompertz curve, provided of course that the units in which time is expressed are the same.

However, when we determine the time required for each tumor to grow from a single cell to one of the fixed points already defined, and multiply this time by the $\alpha$ for each tumor, we find no universal constancy. Instead, the pattern of constancy is similar to that found for the number of doublings from one cell to the computed asymptote for each tumor (Table III).

Hence, whether measured in time units or in terms of the ratio of one cell to the limiting number of cells approached by the tumor in its growth, the tumors included in the present study fall into several consistent groups : (1) all the mouse tumors except the E0771, but including the primary osteosarcomas, have one constant value ; (2) the $R_{39}$ sarcomas have a different constant value ; (3) the Walker tumors have a more variable range of growth ; and (4) single examples of three different tumors have values that lie within the range established for the other tumors.

### DISCUSSION

In the first paper of this series we showed that the growth of a variety of tumors of the mouse, rat and rabbit could be described very well by a Gompertz function (Equation 1). In the present paper our analysis of tumor growth has been extended by making use of the mathematical properties of this growth equation, and several fundamental characteristics of tumor growth have become apparent.

In the first place, although the growth of each of these tumors can be approximated by a Gompertz curve, the region of such a curve occupied by a given tumor is limited, and to some extent is characteristic of the tumor. In general the mouse tumors occupy a region near the inflection point and extend well toward the asymptote, while many of the rat tumors occupy a region well back of the inflection point. Since the amount of retardation characteristic of the Gompertz function increases as growth proceeds toward the asymptote, the rat tumors in general experience much less retardation than do the mouse tumors ; i.e., the growth of the rat tumours resembles simple exponential growth much more closely than does that of the mouse tumors. Conversely, it is because of the fact that the rat tumors show much less retardation that they fit the Gompertz curve so far to the left ; the " fitting " of the data is essentially a fitting of contours.

Secondly, at corresponding points in the growth process, the rat and rabbit tumors have generally passed through many more cell generations than have the mouse tumors, and hence are larger. The time required to double the tumor size is a function of the instantaneous rate of production of new cells in relation to the number of cells already present, and hence varies from tumor to tumor, but is generally greater for the rat tumors than for the mouse and rabbit tumors.

Thirdly, when the growth curve is extrapolated back to a tumor size of one cell, we find plausible values for the time at which each tumor would have had this size. In particular, the computed time at which the primary osteosarcomas would have started growing as a single cell is more than 23 days after the start of exposure to the carcinogen, a time that agrees well with the findings for other carcinogenic processes.

In the fourth place, the number of doublings of size required for a tumor to grow from a single cell to the computed upper limit of growth is essentially constant at one value for all but one of the mouse tumors, and at another value for all examples of the $R_{39}$ sarcomas of the rat. A similar constancy is found for the time required for such growth, when the time scale is normalized. Hence there appears to be a strong tendency for different examples of the same tumor to be characterized by a predetermined range of growth from one cell to a final limiting size, which can readily be computed, but which is not usually reached before the death of the host.

These relations suggest strongly that a tumor implanted into a new host grows as though it were a community of cells derived in that host from a single cell at some definite time before implantation. It grows as though it were a single organism, rather than as a population of dissociated individual cells, each the progenitor of an independent line of tumor cells, as presumably bacteria and other free cells do when inoculated into a new culture medium. This relation suggests further that the host plus tumor represents a new, integrated system of growth whose nature we do not as yet understand.

It is clear, however, that the limitation to tumor growth expressed by the form of the growth curve is not a simple homograft reaction, i.e., it is not due to a uniformity in the time course of the development of an immune response, for two different reasons : in the first place, the primary tumors of this series, the osteosarcomas of Finkel, Bergstrand, and Biskis (1961), fit a Gompertzian pattern of delayed exponential growth as readily as do the transplanted tumors, and secondly, the same proportional decay of the specific growth rate is seen in the normal growth of the organism and of its parts, both embryonic and post-natal (Laird, Tyler and Barton, unpublished data), where a homograft immunity can be ruled out with certainty.

The underlying mechanism of the proportional decay of the specific growth rate in these systems, at the level of cell action and interaction, is not the concern of the present study, but constitutes a subject for investigation in its own right. Cell death has been shown to be one of the normal processes of embryonic growth, in addition to the well-known processes of cell proliferation and cell migration (Glucksmann, 1940). Loss of cells from the generative population to differentiation has also been suggested as the mechanism responsible for retardation in embryonic growth (Weiss and Kavanau, 1957) and in renewal systems (Till, McCulloch and Siminovitch, 1964). If cell death, or removal of cells from the generative to another pool, is the underlying mechanism, such a process can only be one whose magnitude increases exponentially during the growth process, as noted in the first paper of this series (Laird, 1964). The present investigation furnishes no direct evidence for any of these mechanisms, except to allow us to rule out homograft immunity as the cause of the exponential retardation.

### SUMMARY

Our analysis of tumor growth based on the Gompertzian equation

$$W = W_0 e^{(A/\alpha)(1-e^{-\alpha t})}$$

has been extended in the present paper by making use of the mathematical properties of the growth equation.

We find that the region of the Gompertzian curve fitted by each tumor is limited and fairly characteristic for each tumor ; that at corresponding points in the growth curve the rabbit and rat tumors are very much larger than the mouse tumors ; that the time required to double the tumor size at corresponding points on the growth curve are generally greater for the rat than for the mouse and rabbit tumors, except for the primary mouse tumors. Plausible values are obtained for the time at which each tumor would have existed as a single cell, when the growth curve is extrapolated back in time. The range of tumor growth from one cell to the final limiting tumor size is quite constant at one value for nearly all the mouse tumors, and at another value for all examples of the $R_{39}$ sarcoma of the rat.

This unanticipated finding suggests that when a tumor is implanted into a new host it grows as though it is a community of cells derived in that host from a single cell, rather than as a population of independent tumor cells.

The mathematical analysis of the Gompertzian growth curve on which the present study of tumor growth depends was carried out by A. D. Barton of Argonne National Laboratory. The details of these mathematical procedures will be presented in another paper.

This work was supported by the United States Atomic Energy Commission.

### REFERENCES

FINKEL, M. P., BERGSTRAND, P. J. AND BISKIS, B. O.—(1961) *Radiol.*, **77**, 269.
GLUCKSMANN, A.—(1940) *Brit. J. Ophthal.*, **24**, 153.
HORNSEY, S. AND HOWARD, A.—(1956) *Ann. N.Y. Acad. Sci.*, **63**, 915.
KLEIN, G. AND REVESZ, L.—(1953) *J. nat. Cancer Inst.*, **14**, 229.
LAIRD, A. K.—(1964) *Brit. J. Cancer*, **18**, 490.—(1954) *Exp. Cell. Res.*, **6**, 30.
*Idem*, AND BARTON, A. D.—(1961) *J. nat. Cancer Inst.*, **27**, 827.
PATT, H. M. AND BLACKFORD, M. E.—(1954) *Cancer Res.*, **14**, 391.
REISKIN, A. B. AND MENDELSOHN, M. L.—(1964) *Cancer Res.*, **24**, 1131.
REVESZ, L. AND KLEIN, G.—(1954) *J. nat. Cancer Inst.*, **15**, 253.
SCHREK, R.—(1935) *Amer. J. Cancer*, **24**, 807.—(1936*a*) *Amer. J. Path.*, **12**, 525.—(1936*b*) *Amer. J. Cancer*, **28**, 345.
SUGIURA, K. AND BENEDICT, S. R.—(1920) *J. Cancer Res.*, **5**, 373.
TILL, J. E., McCULLOCH, E. A. AND SIMINOVITCH, L.—(1964) *Proc. nat. Acad. Sci., Wash.*, **51**, 29.
TING, T. P.—(1952) *Science*, **116**, 149.
WEISS, P. AND KAVANAU, J. L.—(1957) *J. gen. Physiol.*, **41**, 1.